# A spectral three-dimensional color space model of tree crown health

**DOI:** 10.1371/journal.pone.0272360

**Published:** 2022-10-05

**Authors:** William B. Monahan, Colton E. Arnspiger, Parth Bhatt, Zhongming An, Frank J. Krist, Tao Liu, Robert P. Richard, Curtis Edson, Robert E. Froese, John Steffenson, Tony C. Lammers, Randy Frosh

**Affiliations:** 1 USDA Forest Service, Forest Health Assessment and Applied Sciences Team, Fort Collins, Colorado, United States of America; 2 Team Kapili Services, LLC, Fort Collins, Colorado, United States of America; 3 College of Forest Resources and Environmental Science, Michigan Technological University, Houghton, Michigan, United States of America; 4 Esri, Redlands, California, United States of America; 5 Department of Economics and Geosciences, US Air Force Academy, Colorado, United States of America; 6 School of Forest Science and Management, University of Alberta, Edmonton, Alberta, Canada; Technical University in Zvolen, SLOVAKIA

## Abstract

Protecting the future of forests in the United States and other countries depends in part on our ability to monitor and map forest health conditions in a timely fashion to facilitate management of emerging threats and disturbances over a multitude of spatial scales. Remote sensing data and technologies have contributed to our ability to meet these needs, but existing methods relying on supervised classification are often limited to specific areas by the availability of imagery or training data, as well as model transferability. Scaling up and operationalizing these methods for general broadscale monitoring and mapping may be promoted by using simple models that are easily trained and projected across space and time with widely available imagery. Here, we describe a new model that classifies high resolution (~1 m^2^) 3-band red, green, blue (RGB) imagery from a single point in time into one of four color classes corresponding to tree crown condition or health: green healthy crowns, red damaged or dying crowns, gray damaged or dead crowns, and shadowed crowns where the condition status is unknown. These Tree Crown Health (TCH) models trained on data from the United States (US) Department of Agriculture, National Agriculture Imagery Program (NAIP), for all 48 States in the contiguous US and spanning years 2012 to 2019, exhibited high measures of model performance and transferability when evaluated using randomly withheld testing data (*n* = 122 NAIP state x year combinations; median overall accuracy 0.89–0.90; median Kappa 0.85–0.86). We present examples of how TCH models can detect and map individual tree mortality resulting from a variety of nationally significant native and invasive forest insects and diseases in the US. We conclude with discussion of opportunities and challenges for extending and implementing TCH models in support of broadscale monitoring and mapping of forest health.

## Introduction

Forests cover 31% of the world’s land surface area [1]. They are principally threatened by climate and land-use change, fires, storms, and insects and diseases [2–5]. Trees damaged by these disturbances can pose major management challenges at a multitude of spatial scales, ranging from individual hazard trees in urban to wildland-urban interface (WUI) communities [6] to entire stands in wilderness or other protected areas [7,8], each encompassing many different land uses where management options may be limited or at least varied and complex. Due to these challenges, and to strategize possible management actions, the US and other countries stand to benefit from broadscale monitoring that can detect on an annual or periodic basis at high spatial resolutions where trees suffer compromised health [9].

A large and growing body of research seeks to map forest damage and disturbance using machine learning [10,11] and deep learning [12–14] models trained on high resolution multi- or hyper-spectral imagery. However, most of these models have yet to be scaled up and operationalized over broad extents and diverse damage events, thus limiting practical management applications. If we qualitatively characterize supervised classification models of forest health according to three axes–spatial resolution, spectral resolution, and complexity–new models and methods that target high spatial resolution, low spectral resolution, and low model complexity are needed to bridge this operational gap. High spatial resolution is needed to be able to map individual trees, as well as forest stands, or more generally forests at broader spatial extents [15,16]. Low spectral resolution maximizes the number of different high-resolution imagery sources available for training models, and the damage classes we observe in the visible spectrum are distinct and pronounced [17,18]. Finally, low complexity equates to simple models that favor transferability [19], and further–if based on a single image–do not require standardized imagery from two or more points in time.

Here, we present a new Tree Crown Health (TCH) model that meets the specifications of high spatial resolution, low spectral resolution, and low complexity. We describe TCH workflows, share the training data and code required for generating TCH models, quantify TCH accuracy, and illustrate how results may be used to monitor and map healthy versus damaged crowns. Our methods leverage new and emerging cloud computing platforms for geospatial informatics, including Google Earth Engine (GEE) and Amazon Web Services (AWS), to perform data collection, management, modeling, and analysis. These platforms facilitate the integration and analysis of large data volumes (e.g., high resolution imagery over broad extents) on servers in a cloud computing environment, and they represent the future of geo-big data applications [20,21]. Furthermore, the modeling workflows we share include options that rely solely on software and cloud computing platforms that are freely available.

**Fig 1:**
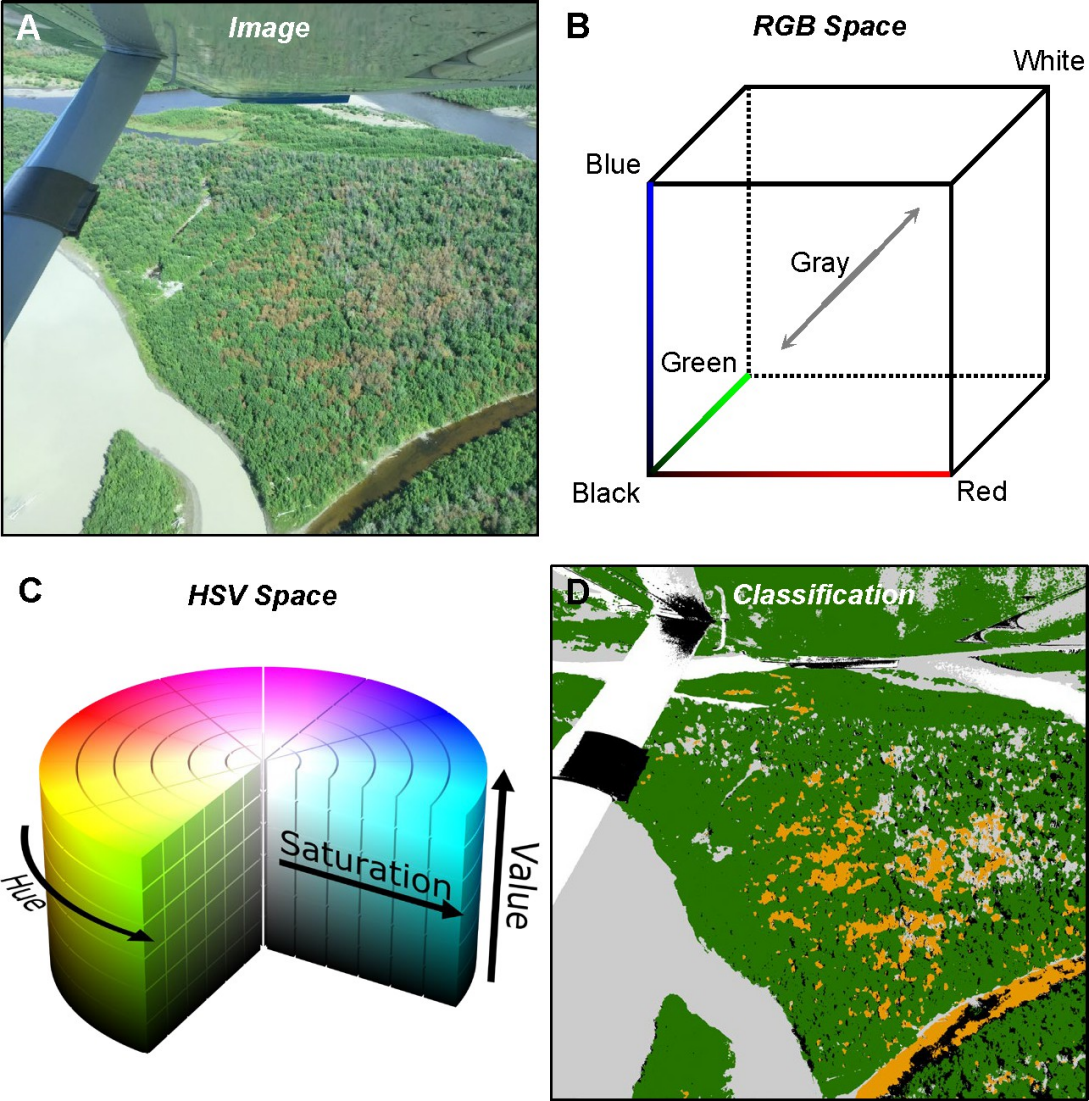
Conceptual model of Tree Crown Health (TCH). (A) Visible damage, as illustrated in the aerial photo on the left, is typically captured by red and gray crowns, whereas healthy crowns appear green, and crown shadows appear dark/black. (B) Photo interpreters perceive these crown color classes and collect training data in red, green, blue (RGB) color space. (C) TCH models are trained after converting RGB to hue, saturation, value (HSV) color space. (D) The classification model is projected back onto the image, where orange = red model class, light gray = gray model class, dark green = green model class, black = shadow or dark object model class, and white = background or areas unclassified by the model. Credits: Photo (A) courtesy of Alaska Division of Forestry; HSV diagram (C) from SharkD, CC BY-SA 3.0 <https://creativecommons.org/licenses/by-sa/3.0>, via Wikimedia Commons.

Conceptually, TCH is based on the ability of humans with normal color vision to interpret general tree crown condition classes in the visible portion of the electromagnetic spectrum. During the growing season, healthy crowns appear in varying forms of green, while damaged, dead, and dying crowns appear as gray, red, or similar colors [17,18], and shadows are evident depending on canopy contiguity and heterogeneity, as well as terrain and the timing and angle of the sensor when the imagery was collected (Fig 1A). Training data in each of these four crown color classes are collected in red, green, blue (RGB) color space (Fig 1B). The training data are evaluated after conversion to the corresponding hue, saturation, value (HSV) color space [22,23] (Fig 1C), to solve for model equation constants that best classify or partition red, gray, green, and shadow crown color classes in the training data (Fig 1D). Hue, or what we think of as color, is generally understood; saturation is how intense the hue is, and value is how dark or light that hue is. Thus, HSV facilitates human visualization and enables computers to statistically separate the variation of greenness (health and species), gray and red (damaged, dead, or dying), and shadow.

The potential applications of TCH models are numerous. Results stand to inform any groups, sites, or activities impacted by hazard trees or snags. These include wildfire incident response teams seeking advanced knowledge of hazardous tree conditions in management zones [24], protected areas requiring management of hazard trees in high human use areas [25], major utilities and other infrastructure (powerline, roads, etc.) impacted by falling snags [26], and developed areas [27], such as WUI communities, where hazard trees present both physical safety hazards as well as fuels for wildfire [28]. Additionally, because snags can provide critical habitat for other species in a forest community [29], TCH models are useful for ecological studies that seek to understand the distribution and location of standing dead trees on the landscape. Results are also potentially useful for studies of long-term disturbance cycles or change events, such as tree mortality resulting from insect outbreaks or climate change.

Damage to trees caused by storms, fire, and other abiotic disturbances are often so severe or intense that a multitude of existing modeling methods and imagery sources already adequately monitor those damage events [30–35]. For this reason, we have focused on applications of TCH models for monitoring damage caused by biotic disturbances; remote sensing has struggled to monitor background mortality as well as tree damage caused by a wide variety of insects and diseases. Many believe that data gaps may be filled by integrating remote sensing with plot or other ground-based observations [9,36], yet ground visits take time and personnel. In the US, over 250 different forest insects and diseases are actively monitored [37]. The interaction of each damage-causing agent and impacted tree species manifests as a different spectral signature that further varies over space and time [17,18]. TCH endeavors to detect and map damage from a wide variety of casual agents in support of broadscale monitoring of forest health.

## Materials and methods

### High-resolution imagery

TCH models are designed to run on any 3-band RGB imagery of high enough spatial resolution for individuals to clearly discern distinct tree crowns and their condition (Fig 1). For our implementation of TCH, we selected imagery from the National Agriculture Imagery Program (NAIP) [38], which in recent years (2012–2019) is typically collected at 60 cm to 1 m resolution. As described further below, TCH uses NAIP in two ways: (1) as a basis for collecting model training data, using photo interpretation techniques; and (2) for projecting the trained TCH models back into geographic space using the RGB band information. Although NAIP is 4-band imagery (RGB + near infrared), TCH models only utilize the RGB bands (unsigned 8-bit integer). Distinct advantages of NAIP, relative to other available sources of high-resolution imagery, are that the images are generally cloud-free (< 10%), collected under reasonably standardized conditions, provide complete coverage of all states in the contiguous US, and are freely accessible. However, NAIP has some disadvantages. It has traditionally been collected for states in alternating years (e.g., Colorado NAIP was collected in 2019 at 60 cm, and in 2017, 2015, and 2013 at 1 m), meaning more ephemeral damage events such as defoliation may be missed. In addition, although it seeks to provide imagery near peak growing season, some images are collected in spring or fall, during times when TCH models cannot be trained (i.e., because of the phenology of tree species and insects and pathogens). Any imagery collected during spring leaf-out or fall senescence, determined by date and visual inspection of the imagery, was excluded from this study. We describe in greater detail, below, how we screened for these, and other issues encountered with NAIP, based on how such issues impacted the collection of training data and model performance.

### Training and testing data

TCH models are a form of supervised classification that are most easily fitted using training data. However, because the training data are used to solve for optimal model constants in equations (see below), a user may optionally evaluate model results using different constants to manually or visually “train” TCH models (the classification in Fig 1D was visually trained). For this study, we collected training data from 190 statewide NAIP imagery datasets acquired from 2012 to 2019, to provide a quantitative assessment of how TCH model classes were distributed in RGB color space. We targeted four RGB color classes: green (healthy crowns), gray (damaged or dead crowns), red (damaged or dead/dying crowns), and shadow (dark areas interspersed between crowns). The green, red, and gray color classes are digitized in accordance with standard training materials that relate “aerial signatures” to tree condition status or health [18].

Training data were collected using photo interpretation techniques, wherein trained individuals visually inspected NAIP imagery and selected representative pixels from each of the four crown color classes (red, gray, green, and shadow) in which to digitize points. Point digitizing and data collection and management were handled using a custom reactive single-page application graphical user interface (GUI) developed and deployed using the JavaScript application programming interface (API) for Google Earth Engine (GEE) [39]. The NAIP Point Digitizing GUI (NPD, S1 Fig) navigated users through a series of four steps to prepare imagery for digitizing and collecting data points using photo interpretation: (1) select the desired NAIP state x year combination; (2) enter digitizer identification and select the ecoregion [40] sample stratum (we used Bailey’s ecoregion sections, defined by ecocodes, which range in size from approximately 6000 to 226000 km^2^); (3) optionally filter NAIP calendar dates to exclude spring or fall imagery when trees were not fully foliated; and (4) set crown color class, enter or digitize points in pixels of that class (delete errors as needed), and export final digitized points to a GEE asset managed feature collection.

Along with NPD and NAIP imagery, digitizers periodically consulted other available recent/historic imagery in Google Earth as an additional guide for digitizing points in Google Earth Engine (Google Earth and Google Earth Engine are separate systems); this was helpful for examining whether canopies were comprised of deciduous vs. coniferous trees, which often have different color signatures of damage, based on leaf-off (winter) imagery, and for examining approximately when damage or mortality occurred in the past. We considered different computer monitor settings and determined that any such variation was small in relation to the inherent color variation within each color class. Additional details and guidelines for digitizing training points, including how areas were located in the imagery (within each state and ecoregion), how pixels were evaluated to determine whether a point should be digitized, what spatial scales were used in digitizing points, etc., are provided in training documents and materials that are included with the NPD code [41].

In each ecoregion stratum (bounded by each state’s NAIP imagery), a minimum sample of 10–15 points was sought for each of the four crown color classes. Note that since NAIP is collected state by state, and neighboring states are often flown in different years and/or by different vendors utilizing different sensors, we could not fully sample ecoregions that spanned state boundaries. Thus, our ecoregional strata were sampled within each NAIP state x year combination. Since the TCH models are trained in spectral space (not geographic space), these samples were intended to capture the spectral range of variation observed in each of the four classes. Some of that variation could be sampled by digitizing clusters of 3–5 points (pixels) per tree crown. In total, we collected 238509 points in 190 unique NAIP state x year combinations across the four crown color classes.

For each NAIP state x year combination and class that had ≥ 50 digitized points, we used 90% of the points for model training and the remaining 10% for model testing; otherwise when a class had < 50 points, we did not subset testing data and instead allocated all points to training. A total of 214845 points were used in training, and 23664 points for testing. Digitized points were checked for quality in the model equations, below. The partitioned NPD data used to train/test TCH models, along with RGB and near-infrared NAIP pixel values associated with each point, are available for download [41].

### Model equations

While the TCH training data described above were collected on maps of RGB images, the color space most familiar to photo interpreters, TCH models are mathematically trained in HSV color space. HSV has been utilized within the remote sensing community for many other applications, such as forest fire recognition, urban area image classification, forest detection, shadow detection, tree cover delineation, wetland classification, and invasive species identification [42–46]. In the case of TCH, the distinct advantage of HSV is that hue (*H*) is a measure of color, saturation (*S*) is a measure of color purity, which when low may be perceived as gray, and value (*V*) is measure of darkness, and these categories conform well to our four model training classes. Thus, for TCH, hue is used to model green and red crown classes, saturation is used to model gray crowns, and value is used to model shadows or dark objects. HSV values in all equations below are scaled from 0 to 1, so that the equations can be applied to imagery of any unsigned bit depth that has been max normalized.

For green and red classes, we first rescale hue so that the maximum of green (*H* = 1/3) or red (*H* = 0 or 1) is 1. Furthermore, we allow for each of the two color classes to retain a non-zero positive value within ± 1/6 of the maximum. For example, rescaled green values are > 0 when hue values are > 1/6 (yellow) or < 1/2 (cyan). Similarly, rescaled red values are > 0 when hue values are > 5/6 (magenta) or < 1/6 (yellow). Thus, the red model class also includes damage associated with hues that tend towards brown, orange, and yellow, as these are also important color signatures of damage [17]. In addition, the green model class includes crowns that also tend towards yellow, for certain tree species may appear yellow-green during the growing season (e.g., quaking aspen, *Populus tremuloides* Michx.). Hues close to yellow are classified red if R > G and green if G > R. In Eq (1) (rescaled green hue, *Hg*) and Eq (2) (rescaled red hue, *Hr*), the rescaled hue values are needed to restrict each of the two model classes to the appropriate portion of HSV color space:

Hg=1−|H−1/3|/(1/6) (1)

Hr=|1/2−H|/(1/6)−2 (2)

Once hue rescaling is performed, the green (*G*, Eq (3)) and red (*R*, Eq (4)) models are simple expressions that rescale the *Hg* or *Hr* values from 0 to a maximum of 1, based on model constants (*c*_*g*_ and *c*_*r*_):

$$G=\left\{\begin{array}{c} (c_{g}{}^{Hg-1})/(c_{g}-1),\;Hg\geq 0\\ 0,\;Hg<0 \end{array}\right..$$ (3)

$$R=\left\{\begin{array}{c} (c_{r}{}^{Hr-1})/(c_{r}-1),\;Hr\geq 0\\ 0,\;Hr<0 \end{array}\right..$$ (4)

Where *c*_*g*_ and *c*_*r*_ are constants for *G* and *R*, respectively, and they are defined for all values ≥ 2.

Gray (*Y*, Eq (5)) and shadow or dark object (*D*, Eq (6)) equations require *S* and *V* as predictors. The constants (*c*_*y*_ and *c*_*d*_), again ≥ 2, determine the rate at which *Y* and *D* decay to 0:

$$Y=(c_{y}{}^{1-S}-1)/(c_{y}-1)$$ (5)

$$D=(c_{d}{}^{1-V}-1)/(c_{d}-1)$$ (6)

Once Eqs (3)–(6) have been solved, final classification is determined by first computing the maximum value:

X=max{G,R,Y,D} (7)

And, assuming *X* exceeds some minimum classification threshold (*t*), where *X* > *t*, then assigning *X* to the appropriate class (*C*):

$$C=\left\{\begin{array}{c} G=X,green\\ R=X,red\\ Y=X,gray\\ D=X,shadow \end{array}\right.$$ (8)

The above equations are valid for all values of RGB, converted to HSV, except in two cases. One is when *G* = *X* and the red and blue values in RGB are equal, or when *R* = *X* and the green and blue values in RGB are equal. In these situations, *G* or *R* operate independently of the other two values in RGB, even if *S* or *V* are very close to 0. This independence of *G* and *R* on the other two values in RGB is not permissible and to correct for it we take the blue value in RGB and decrease it by 1/256 (that adjustment factor is due to NAIP being unsigned 8-bit integer; other bit depths and types would necessitate equivalent adjustment factors). The adjustment factor is applied to RGB values where needed just prior to converting RGB to HSV; all other unaffected RGB values are left unchanged. Eqs (1)–(8) then proceed as described above.

The other special case requiring adjustment is when *S* = 0. That occurs when all three values in RGB are identical, and it represents true gray. However, when *S* = 0, it is independent of *V*. This can result in situations when *V* is very close to 0 (i.e., black) but *Y* = *X* = 1, resulting in shadow or dark objects being classified as gray. To correct this situation, and only in situations where *S* = 0, we recalculate *Y* as Eq (5) minus Eqs (6), (7) and (8) then proceed as described above.

While these are important adjustments to correct for proper behavior in the TCH model, we emphasize that these cases in actual imagery such as NAIP are quite rare.

### Model constants

We solved for optimal model constants (*c*_*g*_, *c*_*r*_, *c*_*y*_, and *c*_*d*_) using the training data and model equations. These constants were optimized for each NAIP state x year combination, meaning we solved for them for all 190 models. The optimization routines were written in R [47] and are available for download [41]. For each combination of state and year with NAIP imagery, we permuted all possible combinations of model constants that included values 2, 5, 10, 20, 30, 40, 50, 60, 100, 1000, 10000, 100000, and 1000000 for *c*_*g*_ and *c*_*r*_, and values 2, 5, 10, 100, 1000, 10000, 100000, 1000000, 10000000, and 100000000 for *c*_*y*_, and *c*_*d*_. In each permutation, we classified each pixel with a training point into one of the four model classes (red, gray, green, or shadow). We then computed a confusion matrix across all samples and calculated model sensitivity (true positive rate, TPR) in each model class. After averaging sensitivities for red, gray, green, and shadow, we selected the optimal model constants based on the highest mean TPR. An additional filter was then applied to eliminate NAIP state x year models where the minimum TPR across the four model classes was < 0.7. This filter was applied because NAIP can vary in quality (e.g., color balancing) for different states and years, and cases where training sensitivity was < 0.7 were generally ones where NAIP quality presented challenges for photo interpreters to accurately and consistently digitize training points into the four classes. Of the 190 NAIP state x year combinations where training data were collected, 167 of those were retained by nature of minimum TPR being ≥ 0.7. The optimal model constants selected using these criteria are available for download, along with the R code to permute and evaluate different sets of constants for use in the models [41].

**Fig 2:**
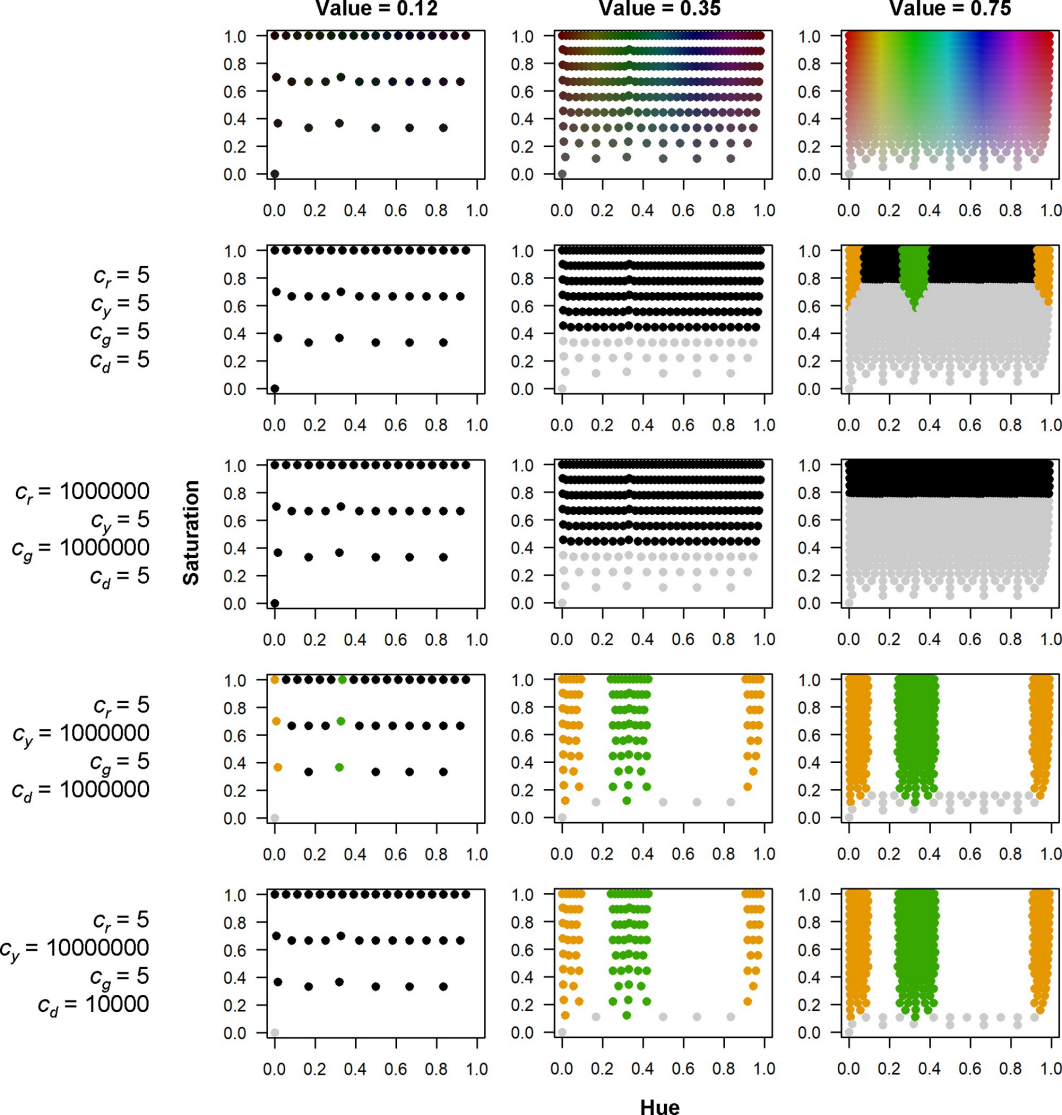
Tree Crown Health (TCH) model classifications in hue, saturation, value (HSV) color space. Plots in top row show color in relation to hue (x-axis), saturation (y-axis), and three levels of value, corresponding to the three columns of plots (*V* = 0.12 [left column], 0.35 [middle column], 0.75 [right column]). Plots in rows two through five (bottom row) show how HSV color space is classified using the TCH model equations and different sets of model constants, shown to the left of each row of plots (orange = red model class [red tree crowns], light gray = gray model class [gray tree crowns], dark green = green model class [green tree crowns], black = shadow or dark object model class [crown shadows], and white = background or areas unclassified by the model [i.e., *X* ≤ *t*, in Eq (8), where *t* = 0.1]). The left and middle columns of plots have fewer points because, at lower values, hue and saturation are compressed into smaller portions of HSV space. The bottom row of plots corresponds to the median optimal model constants across all NAIP state x year combinations, and thus is most representative of the best fit models.

We evaluated a wide range of model constants in our study, to fully explore model parameter space, and because the model constants are critical to fitting models using Eqs (1)–(8) (Fig 2). Nonetheless, certain combinations of constants are often likely unacceptable for partitioning HSV color space into the four model classes, such as when all constants are small (5) or *c*_*g*_ and *c*_*r*_ are large (1000000). Meanwhile, different combinations of constants where *c*_*g*_ and *c*_*r*_ are small (5) and *c*_*y*_ and *c*_*d*_ are large (10000 to 10000000) tend to similarly classify the four model classes, and the classes themselves conform well to human perception of the class colors.

### Model implementation

TCH model code is available for use in the JavaScript API for GEE [39], as well as ArcGIS® Pro [48] as a Raster Function written in Python v.3.7.10; the two versions of code are available for download [41]. While both platforms enable users to run TCH models and generate predictions in raster format, the latter can be deployed in multiple cloud environments using ArcGIS® Image Server–Raster Analytics [49], which allowed us to run and save results for modeling domains at spatial extents that were not permissible in GEE (e.g., entire states), and to seamlessly ingest model results into any other software needed for post-processing, analysis, and visualization. In our case, we deployed the workflow using ArcGIS® Enterprise [50] in Amazon Web Services (AWS, S2 Fig). The GEE and Python scripts referenced above both replicate TCH raster predictions for equations and optimal model constants.

To identify damaged trees as objects (vector polygons or points), we use two additional geoprocessing GUIs deployed in ArcGIS® Pro. The first ingests the raster outputs of ArcGIS® Image Server–Raster Analytics and converts the contiguous groupings (rook moves) of red and gray pixels to polygons, saving them as a feature class in a geodatabase [41]. The second uses the “Shape_Area” geodatabase field created in the previous geoprocessing step to then optionally select polygons of each class (red or gray) based on a size range and save them out as point centroids, constrained to being inside each polygon [41]. The second geoprocessing step is valuable for eliminating model noise from spurious pixels as well as commission errors from non-treed objects, for damaged tree crowns tend to fall within a relatively narrow size range. In our results that utilize these two additional geoprocessing steps, we set the size thresholds in the second step to ≥ 4 m^2^ and ≤ 50 m^2^. We selected these thresholds because they roughly equate to radii of 1 and 4 m, which generally approximate well canopy crown sizes throughout our study areas.

### Model evaluation and testing

For all model classes where a minimum of 5 testing points were available for each of the four model classes (122 NAIP state x year combinations), we calculated overall accuracy and Kappa on these randomly withheld points, using the package “caret” [51] in R [47]. These results provided quantitative assessments of TCH model performance. We performed these assessments once using the optimal model constants for each NAIP state x year combination, and again using median optimal constants across states and years (*c*_*r*_ = 5, *c*_*y*_ = 10000000, *c*_*g*_ = 5, *c*_*d*_ = 10000). The latter was done to evaluate model transferability, by comparing model accuracies when trained using a single set of model constants (i.e., a “global” model across all states and years).

In addition, we selected known damage events caused by forest insects and diseases and examined how well TCH models mapped tree damage for those same areas. Recent damage events from insects and diseases were selected from the Insect and Disease Survey (IDS) [52], and we reference specific damage observations according to the unique “DAMAGE_AREA_ID” in IDS. We do not show the IDS data in our figures primarily because they are collected at a much coarser scale than our TCH models (i.e., the IDS polygons would often fully encompass and extend beyond the figure extents). Although the presence of tree damage (red and gray crowns) cannot be statistically evaluated using IDS, due to the scale mismatches, IDS data provide a useful guide for understanding likely agents causing damage to particular host tree species.

Since TCH does not distinguish between treed vs. non-treed areas, we applied a forest vs. non-forest masking layer derived from the 2016 National Land Cover Database (NLCD) [53]. We created this layer by reclassifying NLCD as forest for values 41 (deciduous forest), 42 (evergreen forest), 43 (mixed forest), and 90 (woody wetlands), while all other NLCD classes were reclassified as non-forest. The non-forest pixels derived from NLCD were at 30 m, and any TCH model predictions occurring within these 30 m pixels were masked out.

## Results

TCH models exhibited high accuracies when assessed using randomly withheld testing data for 122 different models reflecting different NAIP state x year combinations. While minimum values for overall accuracy (0.55) and Kappa (0.41), both for the state of Massachusetts the first year NAIP imagery was analyzed (2012), were rather low, median values for the 122 models were 0.89 (overall accuracy) and 0.85 (Kappa), indicating that most of the models performed well when assessed over all four model classes. Furthermore, the 5^th^ percentile for overall accuracy (0.71) and Kappa (0.60) indicate that 95% of models have measures of performance greater than or equal to these values. The model accuracy results obtained using independent testing data for all 122 models, including overall accuracy, Kappa, and TPR (below), are available in the model constants table [41].

The sources of confusion or error in the models followed clear patterns. Shadow was the class with the lowest TPR based on the testing data (38% of models), followed by gray (29%), red (18%), and green (15%). For models where shadow exhibited the lowest TPR, almost all of the confusion was due to shadow being misclassified as gray (94% of models). Meanwhile, models where TPR was lowest for gray tended to misclassify gray as red (57%), and–conversely–models where TPR was lowest for red tended to misclassify red as gray (68%). This pattern of gray and red confusion is expected based in part on damaged trees tending to fall on a color gradient from red to gray, and in either case both model classes signify damaged crowns. Lastly, models where green was the class with the lowest TPR tended to misclassify green as shadow (89%).

TCH models exhibited comparably high accuracies when assessed using the single set of median optimal model constants (*c*_*r*_ = 5, *c*_*y*_ = 10000000, *c*_*g*_ = 5, *c*_*d*_ = 10000). For the same 122 models that were also assessed using individually trained models, median overall accuracy was 0.90 and median Kappa was 0.86, indicating that a single global model using a standard set of model constants can match model accuracy while also being highly transferable.

When compared to known damage events, TCH models reliably mapped tree damage (red and gray crowns) from a wide variety of regionally and nationally significant forest insects and diseases in the US, where damage events were originally detected and attributed as part of IDS. We show results, below, using a combination of raster model predictions for the four color classes (red, gray, green, and shadow), as well as for red and gray crowns as individual objects (points).

**Fig 3:**
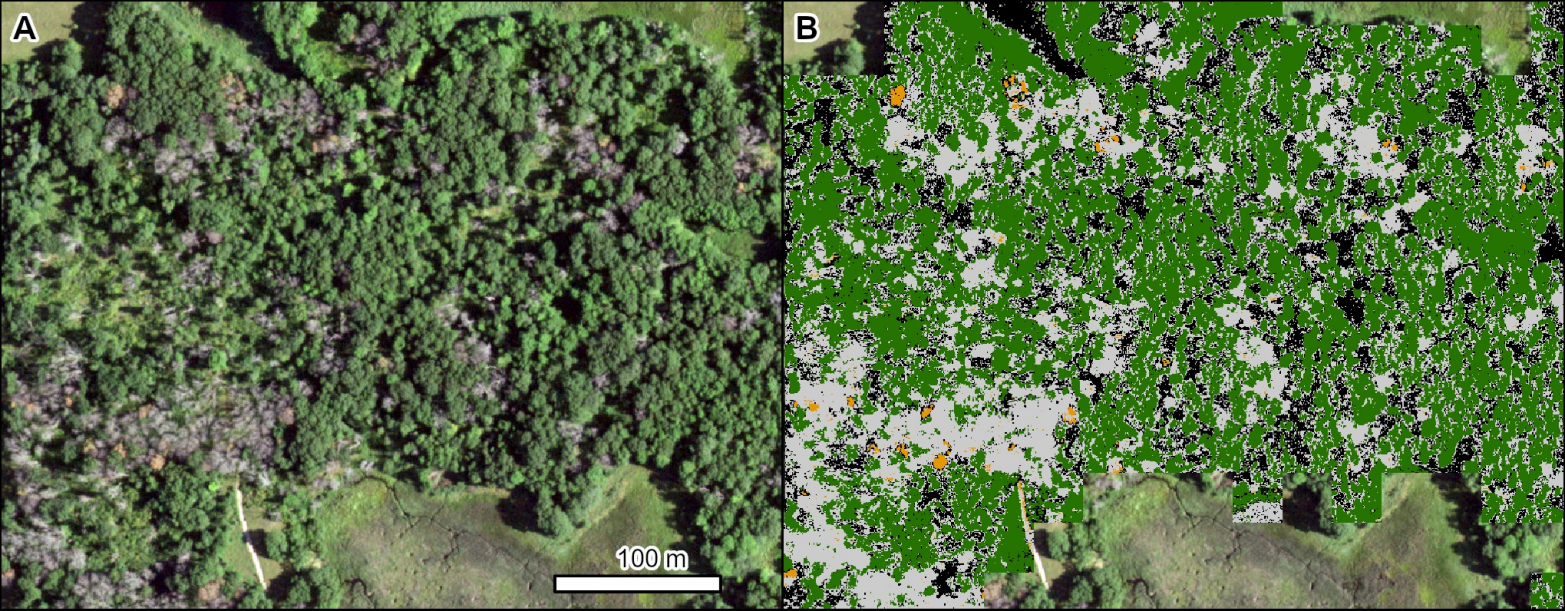
Mortality in a mixed stand of northern pin oak (*Quercus ellipsoidalis* E. J. Hill) and northern red oak (*Q*. *rubra* L.) caused by oak wilt (*Bretziella fagacearum*). (A) NAIP imagery from 2019. (B) TCH model results (orange = red crowns, light gray = gray crowns, dark green = green crowns, black = shadowed crowns). Location: North of Minneapolis, Minnesota. Insect and Disease Survey database DAMAGE_AREA_ID: {e3260d8f-6e10-4eb1-8e51-23e4a3105d7b}. NAIP imagery (public domain) provided by USDA-FSA-APFO.

The first example is oak wilt (*Bretziella fagacearum* (Bretz) Z.W. de Beer, Marincowitz, T.A. Duong & M.J. Wingfield) in Minnesota, north of Minneapolis. Oak wilt is caused by an invasive pathogen, first described in the US in the 1940s, and it has greatly impacted oaks (*Quercus* spp. L.) in the midwestern, northeastern, and portions of the southern US [54]. This example of multiple active pockets of disease in 2019 shows the progression of mortality from orange/red (dying) to gray (dead) phases (Fig 3).

**Fig 4:**
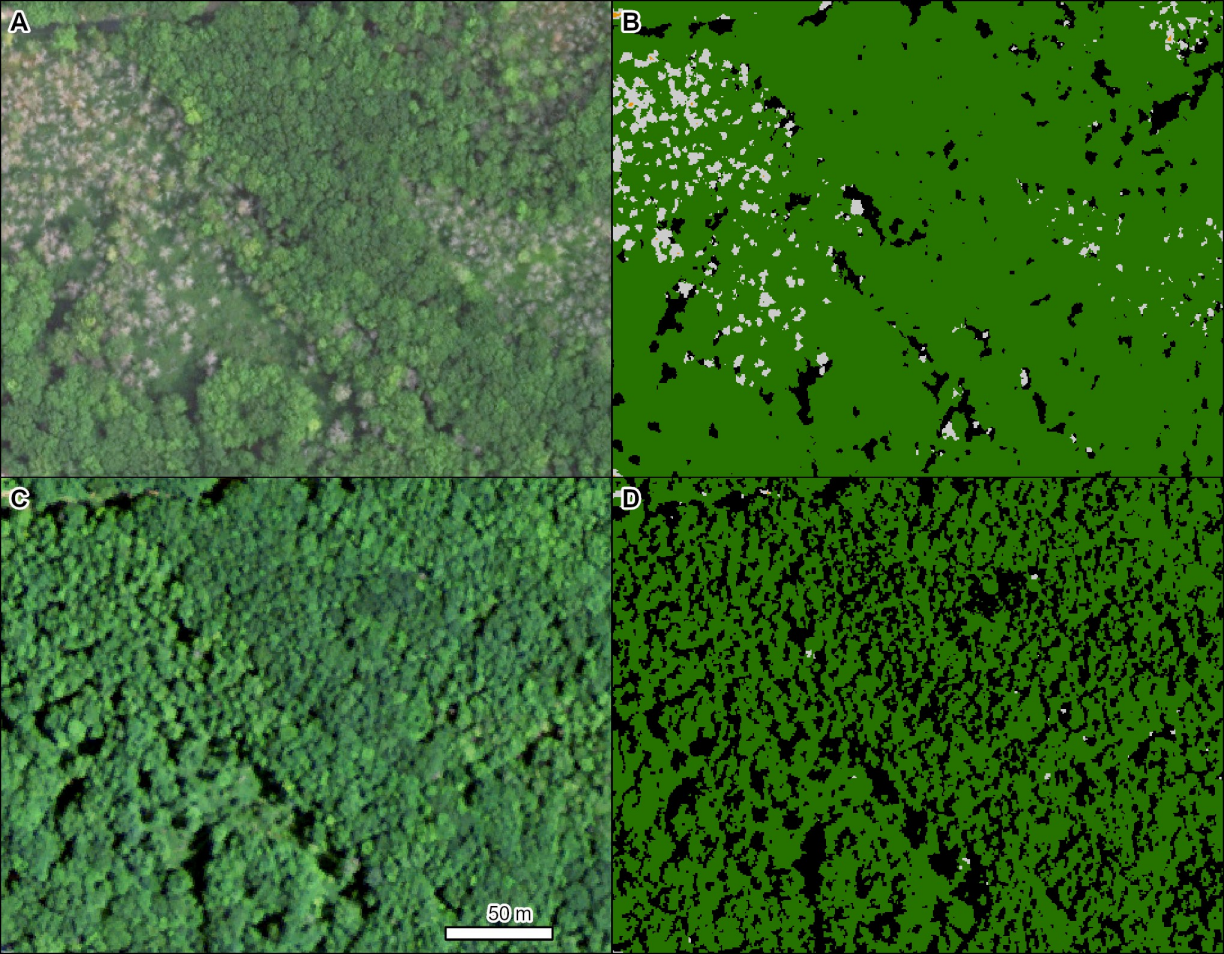
Mortality in a mixed stand of white ash (*Fraxinus americana* L.), green ash (*F*. *pennsylvanica* Marsh), and black ash (*F*. *nigra* Marsh) caused by emerald ash borer (*Agrilus planipennis*). Left panels are NAIP imagery from 2016 (A) and 2012 (C). Right panels (B, D) are the corresponding TCH model results from each year (light gray = gray crowns, dark green = green crowns, black = shadowed crowns). Note: None of the red crown color class existed in this area. Location: Eastern Michigan, near Lake Huron. Insect and Disease Survey database DAMAGE_AREA_ID: {7a2df48e-6e22-45bd-b9cf-b0e8d5a28536}. NAIP imagery (public domain) provided by USDA-FSA-APFO.

Emerald ash borer (*Agrilus planipennis* Fairmaire), an invasive wood-boring beetle that was first detected in the US in southeastern Michigan in 2002 [55], has devastated ash (*Fraxinus* spp. L.) throughout the Midwest. Infested trees often start dying about 4 to 6 years after infestation [56], as illustrated in Fig 4.

**Fig 5:**
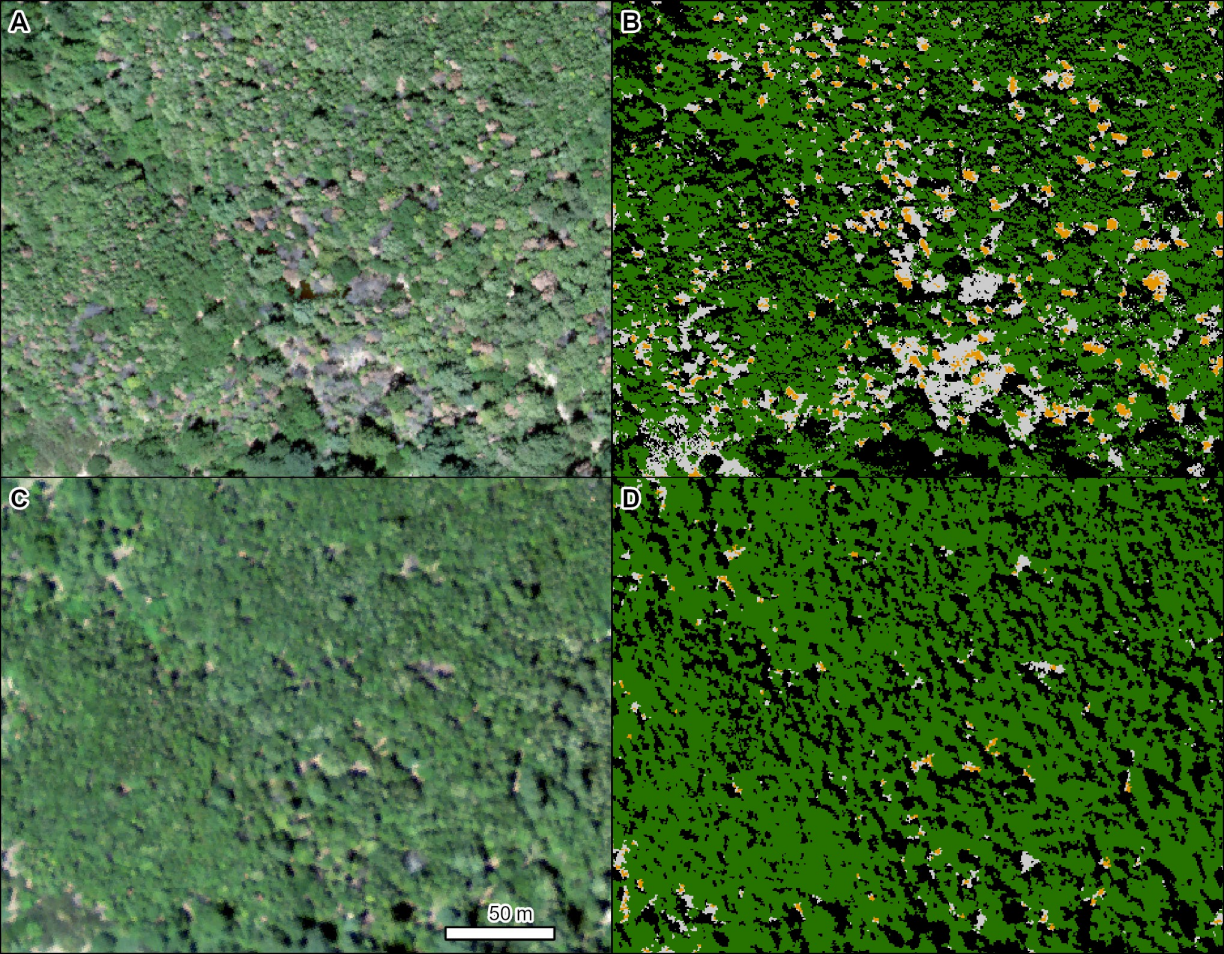
Mortality of tanoak (*Notholithocarpus densiflorus*) caused by sudden oak death (*Phytophthora ramorum*). Left panels are NAIP imagery from 2018 (A) and 2012 (C). Right panels (B, D) are the corresponding TCH model results from each year (orange = red crowns, light gray = gray crowns, dark green = green crowns, black = shadowed crowns). Location: Northern California. Insect and Disease Survey database DAMAGE_AREA_ID: {015c6d5a-ee89-4f6c-a3ea-7799b6401bfc}. NAIP imagery (public domain) provided by USDA-FSA-APFO.

Sudden oak death (*Phytophthora ramorum* Werres, De Cock & Man in’t Veld) is an invasive pathogen first detected in California in the mid-1990s that has since caused significant mortality in tanoak (*Notholithocarpus densiflorus* (Hook. & Arn.) Manos, C.H. Cannon, & S. Oh) and many oaks (*Quercus* spp.) in California and Oregon [57,58]. TCH models detected mortality from sudden oak death over the course of 6 years, from 2012 to 2018 (Fig 5).

**Fig 6:**
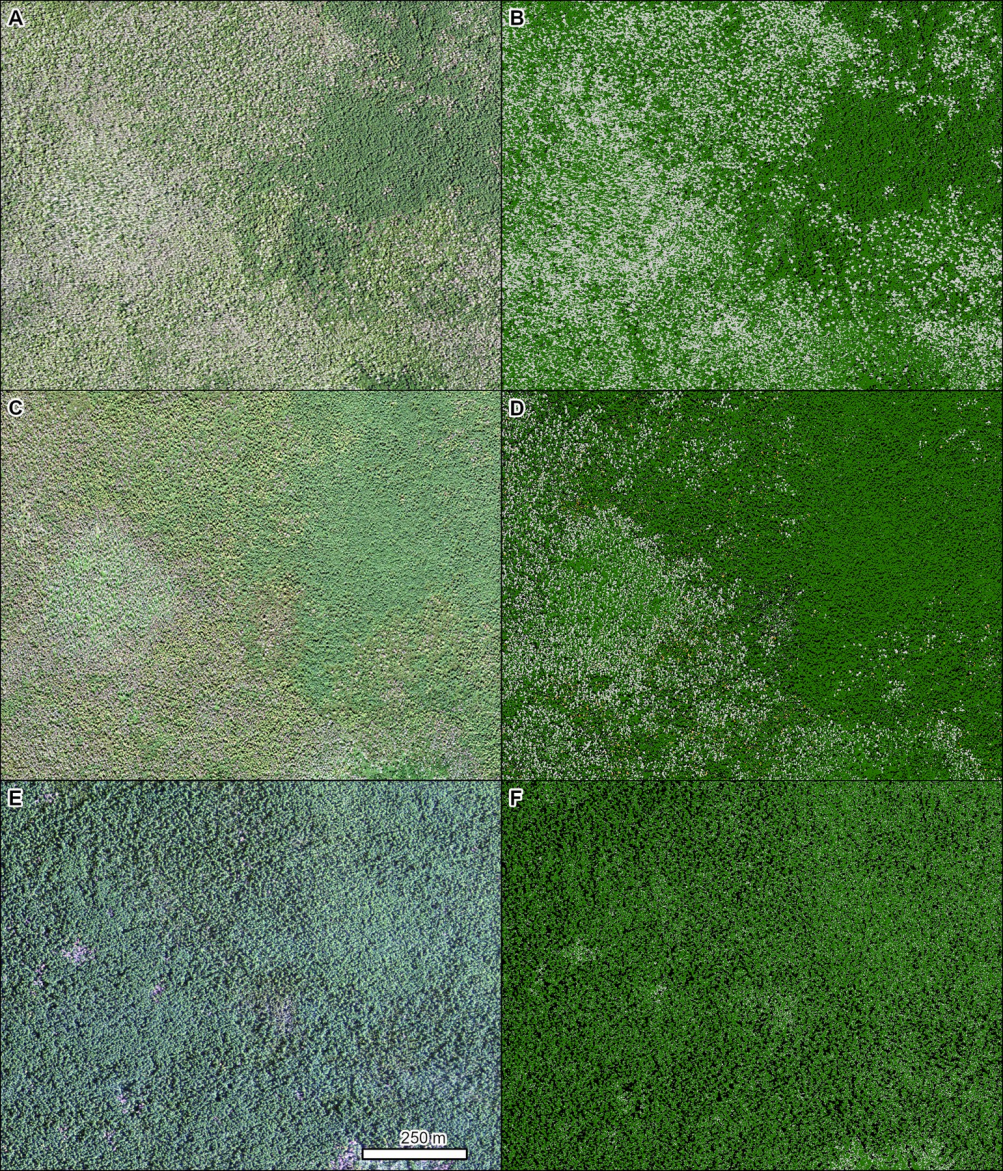
Mortality of tamarack (*Larix laricina*) caused by Eastern larch beetle (*Dendroctonus simplex*). Left panels are NAIP imagery from 2019 (A), 2017 (C), and 2013 (E). Right panels (B, D, F) are the corresponding TCH model results from each year (orange = red crowns, light gray = gray crowns, dark green = green crowns, black = shadowed crowns). Note: The red crown color class is difficult to see at this map scale, but it mostly exists in panel D. Location: Northern Minnesota. Insect and Disease Survey database DAMAGE_AREA_ID: {83accf7a-04b6-4f34-8e8e-4ab2770b42df}. NAIP imagery (public domain) provided by USDA-FSA-APFO.

Eastern larch beetle (*Dendroctonus simplex* LeConte, 1868) is a native bark beetle that can cause extensive and severe mortality in tamarack (*Larix laricina* (Du Roi) K. Koch) [59]. In northern Minnesota, a sharp increase in mortality occurred in 2017, and TCH shows the progression of mortality from 2013 to 2019 (Fig 6).

**Fig 7:**
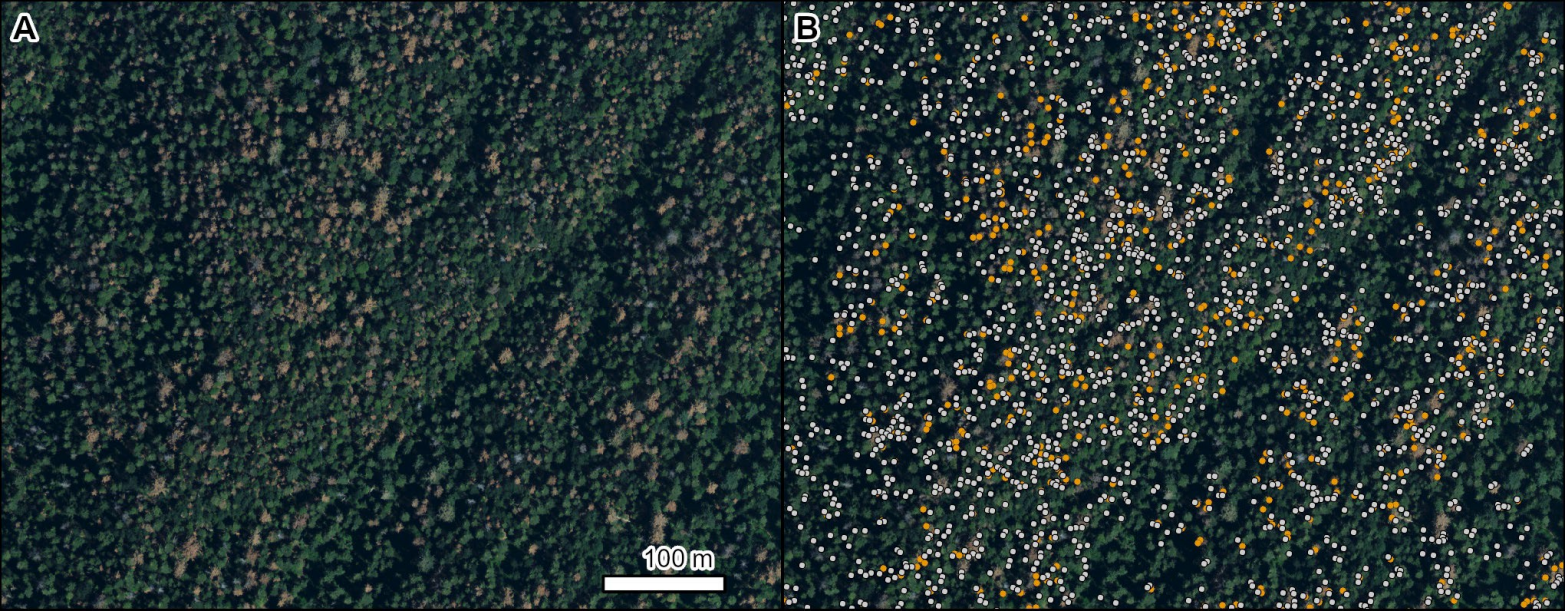
Mortality of ponderosa pine (*Pinus ponderosa*) caused by western pine beetle (*Dendroctonus brevicomis*). Left panel is NAIP imagery from 2016 (A). Right panel (B) shows the damaged tree crowns as points on top of the 2016 NAIP imagery (orange = red crowns, light gray = gray crowns). Location: Sequoia National Forest, California. Insect and Disease Survey database DAMAGE_AREA_ID: 20165031067. NAIP imagery (public domain) provided by USDA-FSA-APFO.

Western pine beetle (*Dendroctonus brevicomis* LeConte, 1876) is another native bark beetle that causes mortality in ponderosa pine (*Pinus ponderosa* Douglas ex P. Lawson & C. Lawson) [60]. In California, likely due in part to recent drought causing tree stress [61], extensive mortality was observed in the Sierra Nevada (Fig 7). This example illustrates opportunities to identify individual red (dying) and gray (dead) tree crowns from the model.

**Fig 8:**
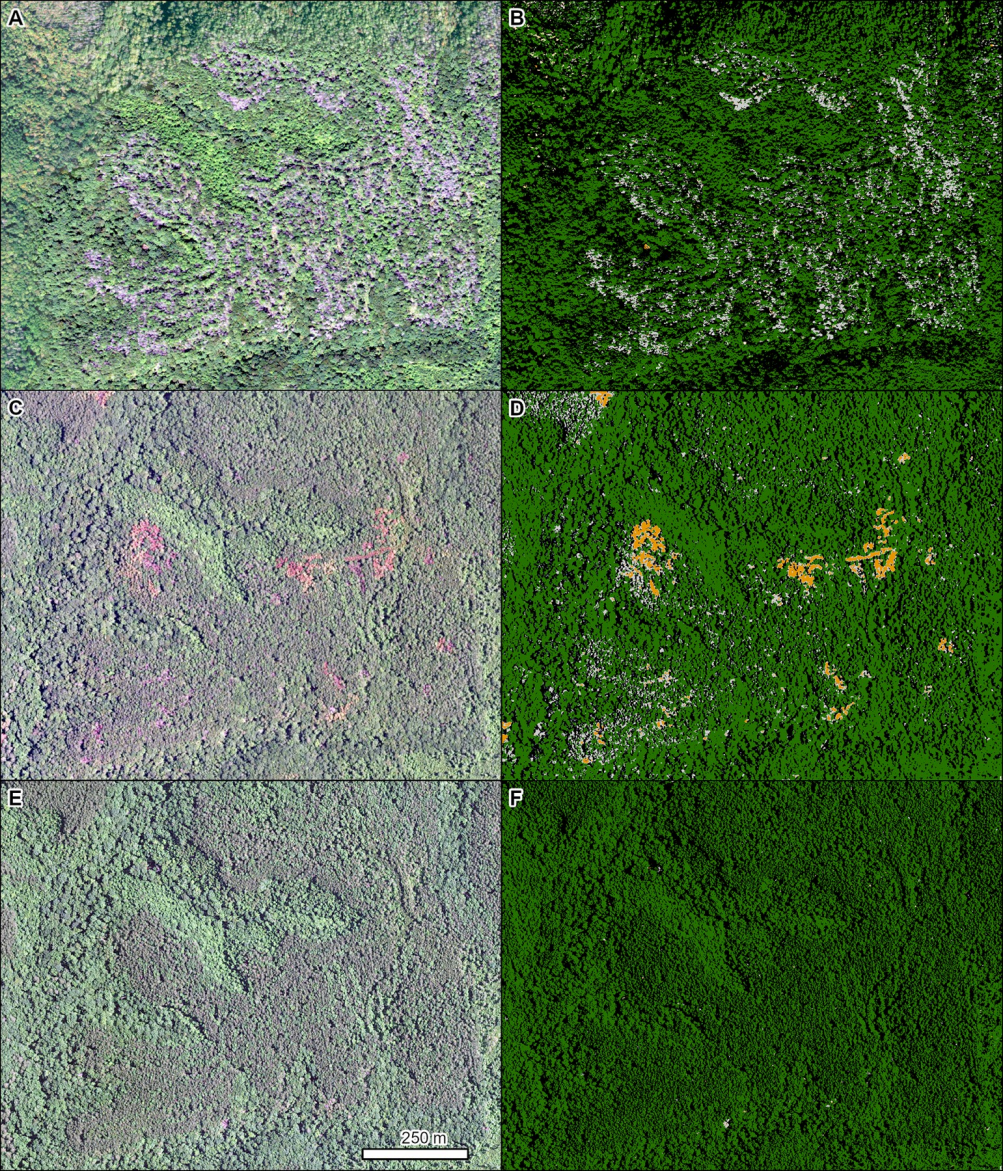
Mortality in a mixed stand of mostly loblolly pine (*Pinus taeda* L.) caused by southern pine beetle (*Dendroctonus frontalis*). Left panels are NAIP imagery from 2019 (A), 2017 (C), and 2015 (E). Right panels (B, D, F) are the corresponding TCH model results from each year (orange = red crowns, light gray = gray crowns, dark green = green crowns, black = shadowed crowns). Location: William B. Bankhead National Forest in northern Alabama. Insect and Disease Survey database DAMAGE_AREA_ID: {d9b4b4b9-855f-4a9f-9d87-87b7c95c211e}. NAIP imagery (public domain) provided by USDA-FSA-APFO.

Southern pine beetle (*Dendroctonus frontalis* Zimmermann, 1868) is a native bark beetle that can cause significant mortality in various *Pinus* spp. L. in the southeastern US [62]. In 2017, a major outbreak of southern pine beetle occurred, an example of which is shown on the William B. Bankhead National Forest in Alabama, pre (2015), mid (2017), and post (2019) outbreak (Fig 8).

## Discussion

Our results demonstrate the ability to monitor and map forest health conditions using mathematically simple TCH models in combination with high spatial and low spectral resolution imagery. TCH thus offers new opportunities for evaluating the health of individual trees to entire stands over broad geographic areas, given that the models only require 3-band (RGB) imagery from a single point in time. Results are useful for informing our understanding of where hazard trees or snags exist on the landscape, which in turn may support other research examining snags as habitat or ecological niches in a community, as well as offer decision making support to resource managers and communities where hazard trees pose safety risks. Results may also contribute to survey and mapping of damage caused by forest insects and diseases, as well as other abiotic disturbances such as storms, fire, and drought. Understanding the distribution and density of standing dead trees, including when they suffered mortality, can help guide timber and salvage operations, which may in turn mitigate fuels and fire risk, and create new opportunities for restoration. Additional opportunities relate to using TCH results to study the environmental correlates of damage distribution and severity, establish standing dead tree baselines for future monitoring in the context of climate change, reconstruct the spread and associated ecological impacts of invasive insects (e.g., emerald ash borer) and pathogens causing disease (e.g., oak wilt), and contribute to inventory and assessment mapping of recent and historical damage.

TCH supports these potential applications by providing both vector and raster data models. Raster results map the extent of healthy green crowns, damaged, dying or dead red and gray crowns, and crown shadows. These outputs enable the estimation of green, red, and gray crown areal extents, while accounting for uncertainty from crown shadows. Following additional post-processing of the raster outputs, vector results of the red and gray crown classes further discern damaged, dying, or dead trees as objects. These outputs enable individual tree counts and trees per acre (TPA) calculations of hazard trees or snags. Since snags often represent older standing dead trees that have lost many of their branches, opportunities may exist to further categorize or distinguish snags from other more recent tree mortality based on gray crown areal extents, where snags have smaller areal extents, compared to recent mortality in trees that still retain many of their branches. Furthermore, the timing of mortality may be determined from TCH models run on imagery from multiple points in time, as illustrated for certain results (e.g., Figs 4 and 5).

Additional work is needed to evaluate the accuracy of TCH models. While the measures of accuracy calculated in this study using randomly withheld testing data were generally quite high, additional assessments using independent testing data are warranted. The figures showing TCH model results in relation to the base NAIP imagery illustrate how TCH may be used to refine maps of damage based on IDS, but the patterns of healthy vs. damaged trees in the images and models can be complex (e.g., Figs 6 and 7). Unfortunately, the IDS data used in our study cannot be statistically compared to our TCH models. IDS data are collected at a radically different (much coarser) spatial scale, and the damage polygons can exhibit low positional accuracy, because they are primarily hand-drawn observations mapped while flying in small aircraft. Rather, as demonstrated in our results, IDS data are primarily useful for understanding generally where and when forest damage occurred, as well as what caused the damage and which tree species were impacted. Unlike IDS, Forest Inventory and Analysis (FIA) data [63] may be used to assess the accuracy of area-based TCH summary statistics, and FIA affords the added opportunity to measure how much understory mortality is occurring that is missed by TCH and other imagery (top down) measures of mortality in the canopy. A quantitative TCH vs. FIA comparison was beyond the scope of this study, but important considerations for future study design include controlling for vertical stratum in FIA, as well as FIA plot revisit frequency, plot sample size, and how those two factors affect FIA area-based estimation of biotic damage events that are often spatiotemporally aggregated or biased.

Variation in TCH model performance is anticipated based on the spatiotemporal scales of damage interacting with the timing and frequency of imagery available to train and project the models. The examples provided in this study all demonstrate the ability of TCH to detect and map tree mortality occurring in trees identified as showing signs of stress. Most trees experience mortality and remain standing dead for multiple years, even many decades in certain environments [64,65]. Thus, our results pertain to damage occurring over long (inter-annual) time scales. However, other damage types such as defoliation occur over comparably short (intra-annual or even intra-seasonal) time scales [66]. These ephemeral damage events may be captured and mapped well by TCH if the available imagery is timed appropriately. Similarly, the spatial scale and spectral signature of damage also affect TCH model results. The tree mortality examples shown here involve entire crowns delineated by multiple contiguous red or gray pixels (i.e., patches). However, other damage types may affect only portions of tree crowns (e.g., branches) or result in signatures that retain more of a green hue (e.g., crown discoloration) [17,18]. In these cases, the spatial resolution of the imagery may be too coarse to detect damage at a sub-crown level, or the signature of the damage may be misaligned with the red and gray models used to detect damage.

Our successes with mapping tree mortality using supervised classification models are not unique to TCH. Other studies have similarly demonstrated the ability to map damage caused by insects or diseases using moderate to high resolution imagery [67–75]. However, most of these studies are highly focused on local and species-specific damage events (e.g., pine mortality from bark beetles [67,69,70]) and are thus limited in their ability to monitor and map damage occurring from the wide variety of insects, diseases, and other abiotic factors affecting forests. In addition, previous studies have often relied on high resolution multi- or hyper-spectral imagery collected for special purposes over relatively small areas (e.g., [10,74]). Methods that rely on such specialized imagery further restrict the areas over which monitoring and mapping of forest health conditions may occur, and they require advanced knowledge of remote sensing to conduct analyses using the specialized imagery. Meanwhile, other studies that utilize existing and readily accessible satellite imagery covering all or most of the world’s surface (e.g., [8,33,66]) do not currently have a high enough spatial resolution to map individual tree crowns. We acknowledge that these and additional remote sensing studies of forest damage are difficult to compare, due to variation in both data and methods. Additional work is required to benchmark various forest health remote sensing modeling techniques while controlling for imagery source, to evaluate which combinations of modeling technique and imagery are best suited to detecting and mapping different damage events.

Our results demonstrate that TCH applied to NAIP or other comparably high resolution RGB imagery offers opportunities to monitor and map forest damage caused by a variety of agents impacting different tree species over broad geographies. Hence, it is a novel method that stands to advance the use and adoption of remote sensing data and technologies in the field of forest health. Nonetheless, additional research is warranted to overcome several key challenges with broadscale implementation, as well as integration with other existing methods that may improve the accuracy and utility of TCH. One key challenge relates to constraining TCH model predictions to treed areas. TCH does not model what is treed but rather the color or condition status of tree crowns. Interpreting TCH results thus requires a separate tree model or mask to restrict model predictions to treed pixels. In this study, we used a simple tree mask derived from NLCD [53], but that mask is at a spatial resolution of 30 m, which is extremely coarse compared to the meter to sub-meter resolution of the imagery used in TCH models. Canopy height models developed from LiDAR (Light Detection and Ranging) could complement TCH models by constraining model predictions to individual tree crowns.

**Fig 9:**
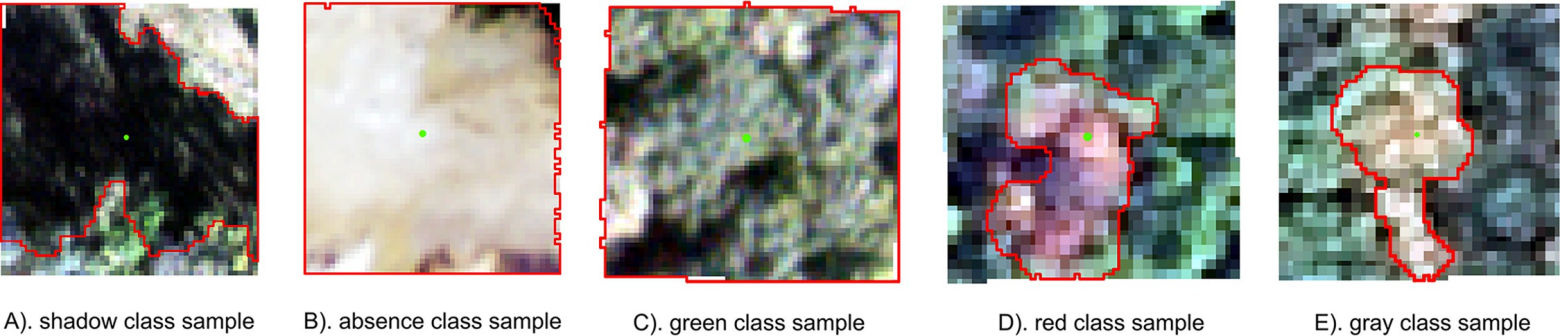
NAIP image chips and labels. A) shadows (South Dakota 2018), B) absence (non-treed areas, Minnesota 2019), C) green healthy crowns (Oklahoma 2019), D) red damaged crowns (Alabama 2017), and E) gray damaged crowns (Virginia 2012). The chips are 59 x 59 pixels in size, and the green points in the center of each chip are the NAIP digitized points collected using the NPD tool in Google Earth Engine, for each respective color class. Red outlines show the results of the algorithm used to “grow” points into patches, with patch labels determined from the color classes of the digitized points. NAIP imagery (public domain) provided by USDA-FSA-APFO.

Deep learning models also show promising results for mapping tree crowns in RGB color space [13,76–79], especially when large labeled datasets like the TCH samples are available, since such models are often limited by the number and quality of labels that are used in model fitting [80]. Future research applying deep learning models to NAIP, or imagery at a comparable spatiotemporal resolution, could greatly improve TCH applications. However, deep learning models usually require two-dimensional image patches as input, while TCH samples are points. One area of future work is to use a region growing algorithm to expand each point to the surrounding homogeneous pixels to convert the point data into image patches, which can then be utilized by deep learning models for model training and prediction. We are exploring the use of a point-to-patch label generation algorithm [81] using TCH training points, and preliminary results suggest these methods may allow us to derive a large number of image chips and labels for use in training deep learning models (Fig 9).

In many instances it is important to know more than where damaged trees exist, but also the tree species impacted, cause of damage, and damage type. The TCH models presented here are unable to assess or predict any of these factors and the examples we show in results were all based on known damage events documented in IDS [52]. IDS data include information on damage causing agent, host tree species, and damage type, enabling us to infer these attributes for TCH results paired with IDS dates and locations. However, for TCH to support real-time operations where such details are needed, additional models or observations would be required to label or attribute TCH model predictions. Other remote sensing studies of forest health that are agent and host specific (e.g., [71,73,74]) could complement TCH results and offer insights on these additional attributes. Alternatively, attributes could be derived by datamining IDS or other existing forest health observational data sources [82,83], as well as information on host tree species [63,84], to probabilistically infer data labels at moderate spatial resolutions. Such integration of remote sensing with other traditional forest health survey data stands to play an important role in reducing risk exposure (i.e., of field surveyors) while also facilitating new and varied approaches to conducting surveys when traditional field activities are seriously curtailed, such as what occurred during the COVID-19 pandemic [85]. Furthermore, TCH results stand to complement existing IDS survey data by offering opportunities to refine or improve estimates of damage location (i.e., mapping damage more precisely and accurately) and intensity (e.g., TPA).

While our demonstration of TCH models is limited to a single source of imagery (NAIP), TCH models are capable of being run on any high spatial resolution RGB imagery. Opportunities exist to apply TCH models to other real-time high spatial resolution satellite imagery, including WorldView or other imagery from companies such as Planet Labs. However, these applications come with challenges, including procuring or gaining access to proprietary imagery and obtaining training data. The latter may be overcome by the high transferability we found with our TCH models trained using NAIP. Given how NAIP has variable observation geometries, we did not expect the global models trained using median optimal model constants to perform as well as the models trained separately for each NAIP state x year combination. The fact that the global model performed as well indicates that TCH models are highly transferable, and further that the model constants are robust to a range of values. These factors suggest that TCH models may be manually or visually trained on representative RGB images from alternative sources (e.g., as illustrated in Fig 1 for an aerial photo), without having to necessarily collect new training data.

To facilitate additional study and research, we share all TCH data and code from this study. Our desire is to encourage others to expand and transcend what we have developed. Using the materials we provide, opportunities exist to collect new training data, set baselines for what healthy, dying, and dead trees could look like in RGB and HSV space, derive new optimal TCH model constants, train and project new and alternative TCH models, and explore the utility of TCH results as data inputs for other more complex models. We contribute these resources to the scientific community with hope that they advance research and development of remote sensing applications to monitoring and managing forest health.

## Conclusion

We demonstrate the ability to monitor and map damage and mortality occurring in individual tree crowns over broad extents using simple supervised classification models trained on high spatial resolution three band (red, green, blue) imagery. The results of these Tree Crown Health (TCH) models applied to imagery provided by the United States Department of Agriculture, National Agriculture Imagery Program (NAIP), showed high accuracy and transferability when assessed over 167 different years and states in the contiguous US. TCH models show promise for operationalizing remote sensing monitoring of forest health conditions and hazard trees over large landscape scales. Furthermore, the training data and code provided as part of this study offer opportunities for extending the TCH models using newly collected training data, new imagery, and new modeling methods, such as models developed using machine and deep learning techniques. Two key challenges lie in quantifying the accuracy of TCH models using independent observational data on forest health conditions and improving our ability to mask treed vs. non-treed areas at sub-meter resolutions.

## Supporting information

S1 Fig NAIP Point Digitizing (NPD) graphical user interface (GUI) in Google Earth Engine (GEE). The GUI navigates photo interpreters through a series of 4 steps that prepare the GEE map interface for digitizing training data in each of the four crown color classes (red, gray, green, and shadow; figure example shown for gray; map portion of GUI not shown).
(TIF)

S2 Fig Conceptual diagram of the Amazon Web Services (AWS) architecture used to deploy Tree Crown Health (TCH) modeling workflows using ArcGIS® software solutions. The system is founded on ArcGIS® Enterprise, which manages work and connections between ArcGIS® Pro and ArcGIS® Image Server, which runs Raster Analytics, as well as connections to storage (file server and s3). The names on the boxes (c5.2xlarge, m5.xlarge, r5.4xlarge, and m5.large) indicate the AWS instances (server specifications) used.
(TIF)
