# Supplementary figures and images for: A spectral three-dimensional color space model of tree crown health
Source: PLoS One. 2022 Oct 5;17(10):e0272360. doi: 10.1371/journal.pone.0272360 (PMC9534400; doi:10.1371/journal.pone.0272360)

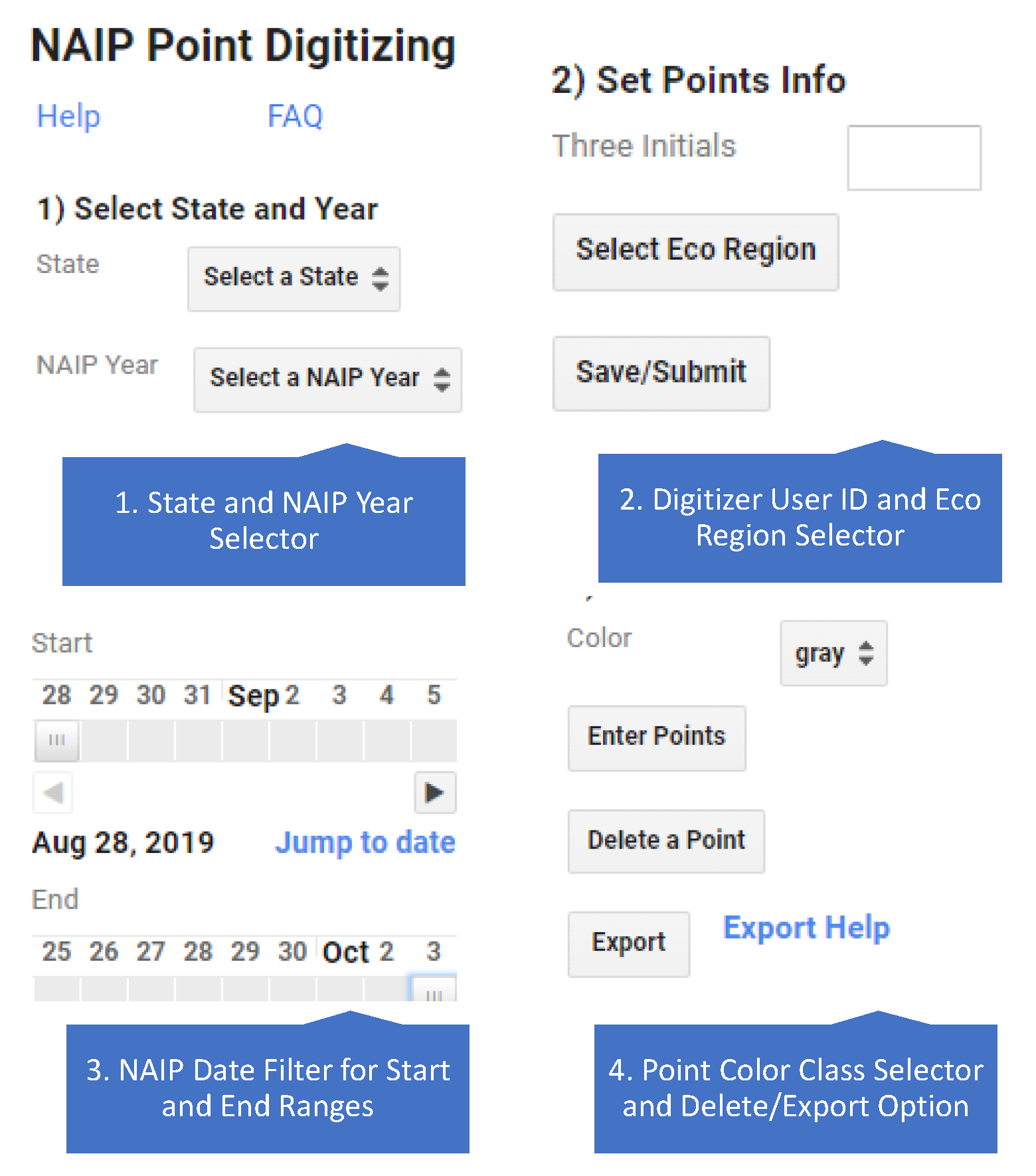

Supplement: S1 Fig — The GUI navigates photo interpreters through a series of 4 steps that prepare the GEE map interface for digitizing training data in each of the four crown color classes (red, gray, green, and shadow; figure example shown for gray; map portion of GUI not shown). (TIF) [file pone.0272360.s001.tif]

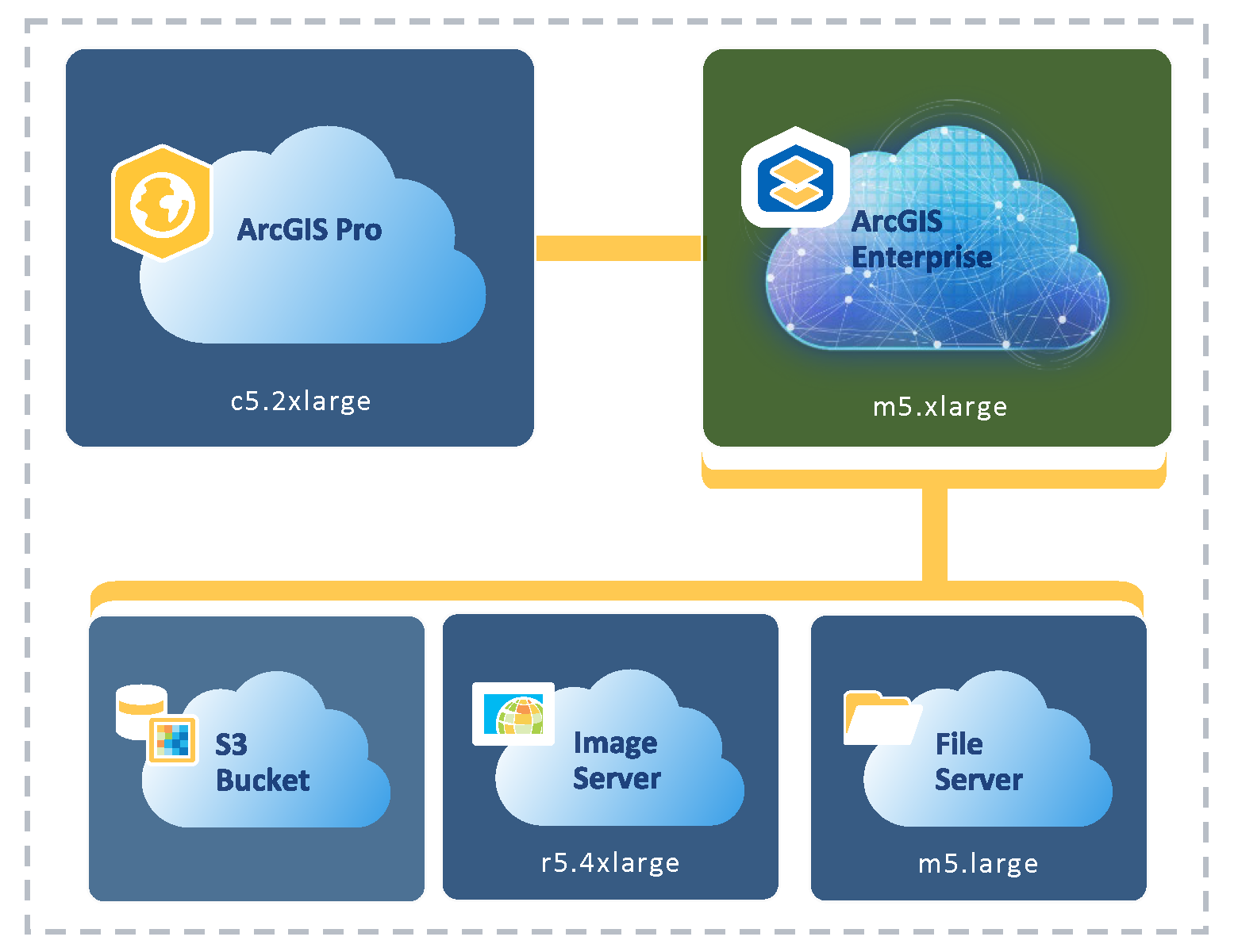

Supplement: S2 Fig — The system is founded on ArcGIS® Enterprise, which manages work and connections between ArcGIS® Pro and ArcGIS® Image Server, which runs Raster Analytics, as well as connections to storage (file server and s3). The names on the boxes (c5.2xlarge, m5.xlarge, r5.4xlarge, and m5.large) indicate the AWS instances (server specifications) used. (TIF) [file pone.0272360.s002.tif]
